# Advances in the Properties of Incomptine A: Cytotoxic Activity and Downregulation of Hexokinase II in Breast Cancer Cell Lines

**DOI:** 10.3390/ijms241512406

**Published:** 2023-08-03

**Authors:** Angel Giovanni Arietta-García, Fernando Calzada, Israel Ramírez-Sánchez, Elihú Bautista, Normand García-Hernandez, Rosa María Ordoñez-Razo

**Affiliations:** 1 Unidad de Investigación Médica en Genética Humana, UMAE Hospital de Pediatría 2° Piso, Centro Médico Nacional Siglo XXI, Instituto Mexicano del Seguro Social, Av. Cuauhtémoc 330, Col. Doctores, Mexico City CP 06725, Mexico; angelo_arietta@hotmail.com (A.G.A.-G.); normandgarcia@gmail.com (N.G.-H.); 2 Unidad de Investigación Médica en Farmacología, UMAE Hospital de Especialidades, 2° Piso CORSE, Centro Médico Nacional Siglo XXI, Instituto Mexicano del Seguro Social, Av. Cuauhtémoc 330, Col. Doctores, Mexico City CP 06725, Mexico; fercalber10@gmail.com; 3 Sección de Estudios de Posgrado e Investigación, Escuela Superior de Medicina, Instituto Politécnico Nacional, Mexico City CP 07738, Mexico; israel.ramirez14@hotmail.com; 4 CONAHCYT—Consorcio de Investigación, Innovación y Desarrollo para las Zonas Áridas, Instituto Potosino de Investigación Científica y Tecnológica A.C., San Luis Potosí CP 78216, Mexico; francisco.bautista@ipicyt.edu.mx

**Keywords:** incomptine A, breast cancer cells, cytotoxic activity, Hexokinase II

## Abstract

Breast cancer treatments are limited by the cancer subtype and its selectivity towards tumor cells, hence the importance of finding compounds that increase the survival of healthy cells and target any subtype. Incomptine A (IA) is a sesquiterpene lactone with demonstrated cytotoxic activity. In this study, through *in vitro* assays, it was observed that IA has similar cytotoxic activity between the subtypes triple negative, HER2+, and luminal A of the breast cancer cell lines. IA cytotoxic activity is higher in cancer than in nontumorigenic cells, and its selectivity index for cancer cells is more than that of the drug doxorubicin. Molecular docking and its *in silico* comparison with the 2-Deoxyglucose inhibitor suggest that IA could bind to Hexokinase II (HKII), decreasing its expression. Since we did not find changes in the expression of the glycolytic pathway, we suppose that IA could affect the antiapoptotic function of HKII in cancer cells. The IA-HKII union would activate the voltage-gated anion channel 1 (VDAC1), resuming apoptosis. Therefore, we suggest that IA could be used against almost any subtype and that its cytotoxic effect could be due to the reactivation of apoptosis in breast cancer cells.

## 1. Introduction

Breast cancer is the worldwide most common disease in women since it represents 12% of new cancers in addition to high mortality rates in women [1]. Breast cancer can be classified into different molecular subtypes based on the expression of the receptors that cover the surface of the cancer cells. These proteins are elemental in sending signals for the cells to grow and divide. The different types of breast cancers are identified, then, by the presence or absence of the receptors found on the surface of the cells: the estrogen receptor (ER), the progesterone receptor (PR), and human epidermal growth factor receptor 2 (HER2) [2]. The treatment is determined depending on the presence or absence of these receptors; therefore, the strategy will be different depending on the type of cancer in question. Despite the advances in targeted therapies against breast cancer, the drugs used often have the problem that they do not have the same efficacy for any stage and/or type of breast tumor nor are they capable of reducing cellular resistance since it is difficult to find adequate concentrations for the chemotherapy to be effective. In addition, they have various adverse effects on patients, ranging from hair loss, fatigue, and pain to cardiotoxicity and hepatotoxicity [3]. Consequently, today, the priority challenge is to discover new therapeutic agents that are more effective and that can be used for any subtype of breast cancer. A compound that may be a candidate is incomptine A (IA). IA is a heliangolide-type sesquiterpene lactone isolated from the leaves of the plant *Decachaeta incompta* (D.C.) that has been shown to alter the expression of the proteins involved in glucose metabolism (enolase, aldolase, and pyruvate synthase) in the trophozoites of *Entamoeba histolytica* [4]. *In vitro*, *in vivo*, and *in silico* models have evidenced that IA has antiproliferative and anticancer activity, that it regulates NF-κB expression, that it induces apoptosis, that it produces reactive oxygen species (ROS), and that it induces the differential protein expression of the glycolytic pathway in non-Hodgkin lymphoma cell lines [5,6]. Additionally, IA showed a dose-dependent cytotoxic effect in the leukemia cell lines HL-60, K-562, and REH [7].

Until now, no studies have been carried out to analyze the effect of AI on breast cancer, but since breast cancer cells have a highly glycolytic metabolism that allows them to have accelerated growth, survival, and proliferation [8], they could be candidates for treatment using IA, which, as already mentioned, can deregulate glycolytic proteins and has a cytotoxic effect on other cancers.

In aerobic glycolysis, hexokinase (HK) is the first rate-limiting enzyme, and it catalyzes the first step in a glucose molecule’s phosphorylation to glucose-6-phosphate from ATP. The five isoforms of hexokinase are structurally similar but show tissue-specific expression [9,10]. Of these isoforms, hexokinase II (HKII) is overexpressed in breast cancer, contributing to the progression, maintenance, and abnormal elevation of glycolysis [11]. Furthermore, HKII is involved in the apoptosis inhibition of cancer cells since it can anchor to voltage-gated anion channel 1 (VDAC1) in the mitochondrial outer membrane (MOM) to exchange ADP for ATP in the mitochondria. This association allows HKII to gain “preferential access” to the ATP produced by the mitochondria while inhibiting apoptosis through the formation of mitochondrial permeability transition pores (mPTP) [12]. Therefore, the VDAC1-HKII junction may be an interesting target for the generation of new treatments aimed at enabling apoptosis.

With respect to the above information, we consider it extremely important to evaluate the antiproliferative effect of IA in breast cancer cells and to analyze its interaction with HKII related to cell death. In the present study, we demonstrated that IA exhibits substantial cytotoxic activity against breast cancer cells and suggest that its mechanism is through its binding to HKII. We also observed that IA caused cytotoxicity in a similar way in all the breast cancer lines tested regardless of the molecular type of cancer, making it a candidate for treatment against any type of breast cancer. 

## 2. Results

### 2.1. Cytotoxic Activity of Incomptine A (IA)

**Figure 1:**
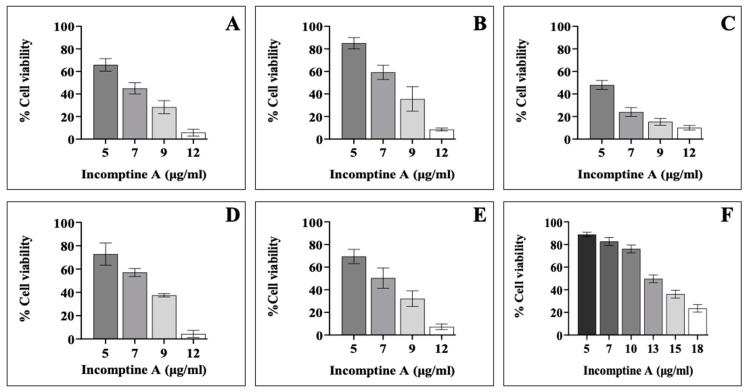
Cytotoxic activity of IA in breast cell lines. Graphs show inhibition of cell growth caused by IA at different concentrations in each cell line and no tumorigenic cell after 24 h of incubation (assays were made in triplicate). The graphs indicate the breast cancer cell lines analyzed as follows: 4T1 (**A**), MDA-MB-231 (**B**), SK-BR-3 (**C**), T-47D (**D**), MCF7 (**E**), and nontumorigenic MCF10A (**F**). These data were used to calculate the CC_50_ of each cell line.

The cytotoxic activity of IA in all the cancer cell lines used in this study was evaluated. The 4T1 cell line (triple-negative mouse breast cancer), MDA-MB-231 (triple-negative human breast cancer), SK-BR-3 (overexpressed HER2 breast cancer), T-47D (luminal A human breast cancer), MCF7 (luminal A human breast cancer), and the nontumorigenic MCF10A cell line (epithelial cell line from mammary gland) were used. To compare the cytotoxic effect of IA, we included the dichloromethane extract from the aerial parts of *Decachaeta incompta* (DEDi), and as positive control, we included the drug doxorubicin (this intersperses into the DNA bases, and through steric obstruction, it inhibits topoisomerase II and the synthesis of DNA or RNA, inducing cell death). Cytotoxicity curves were made from different concentrations of IA (5–18 µg/mL) over 24 h, and the cytotoxic concentration 50 (CC_50_) was determined for each cell line (Table 1 and Figure 1). To obtain the CC_50_ of DEDi and doxorubicin, the concentrations from the cytotoxicity curves were 25–100 µg/mL each (Table 1).

The CC_50_ of IA was much lower compared with DEDi and doxorubicin. The average CC_50_ for IA in the breast cancer lines, obtained from the values in Table 1, was 6.78 µg /mL, whereas, for DEDi and doxorubicin, it was 50.38 and 57.76 µg/mL, respectively. In the search for new treatments against breast cancer, an important factor is that the drug can be used indistinctly against any subtype. The results show that IA had similar CC_50_ values in all the breast cancer lines in this study, contrasting the data obtained with the drug doxorubicin, which varied greatly between them, and, even more, affecting MCF10A (the nontumorigenic line). Therefore, IA acts with a lower concentration and more uniformly regardless of the type of breast cancer. On the other hand, a higher concentration (CC_50_ = 13 µg/mL) of IA was necessary to carry out cytotoxic activity in the nontumorigenic cells (MCF10A), given that it required more than twice the concentration used in the cancer cells, which shows some specificity towards cancer cells and a lower cytotoxic effect on nontumorigenic cells (Table 1). The same was observed when IA was compared with DEDi, given that DEDi did not have a better effect than the compound IA. Although it had cytotoxic activity, it was not as uniform as IA, it required a higher concentration in the breast cancer cell lines, and it had greater cytotoxicity towards MCF10A (Table 1).

### 2.2. Specificity of IA for Breast Cancer Cells

**Figure 2:**
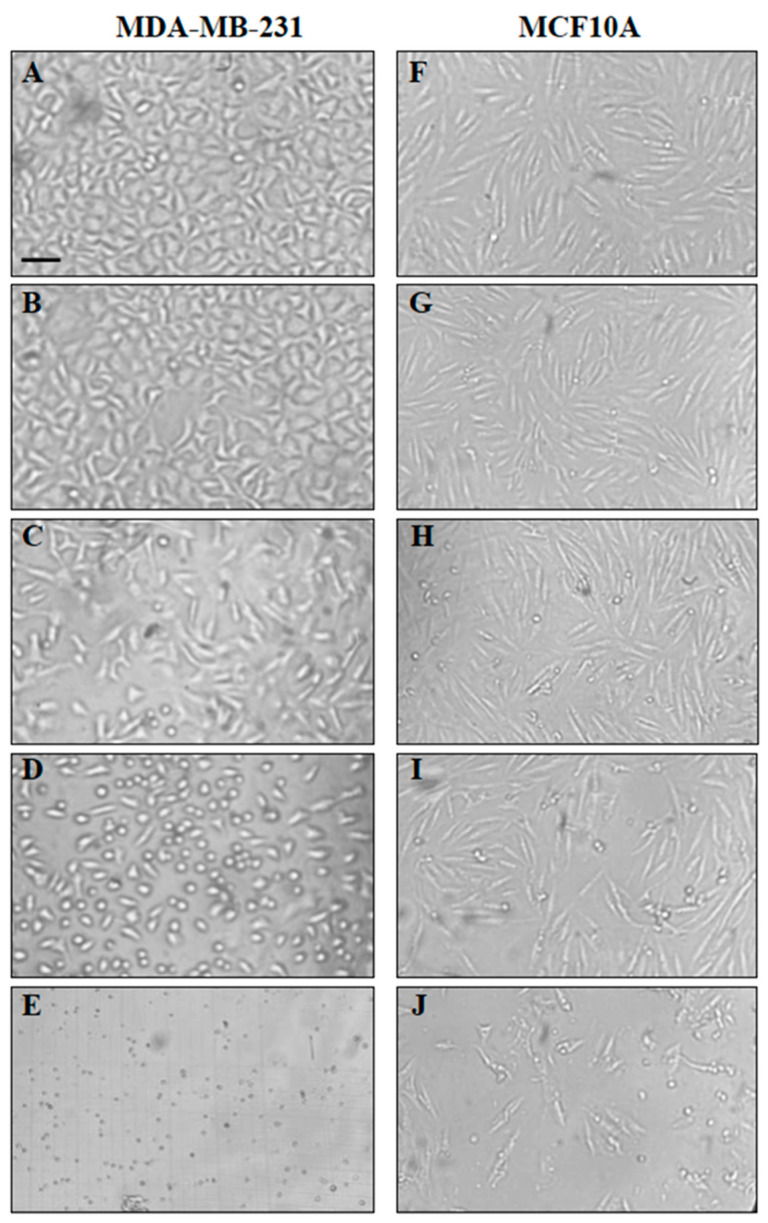
Specificity of IA towards MDA-MB-231 and MCF10A. Cell were treated with different concentrations of IA over 24 h and were observed under invert microscope. (**A**) The MDA-MB-231 cells (DMSO 1% vehicle) and (**B**–**E**) images that show 5, 7, 9, and 12 µg/mL, respectively, of IA. (**F**) The MCF-10A cells (DMSO 1% vehicle) and (**G**–**J**) images that show 5, 10, 13, and 18 µg/mL, respectively, of IA.

As shown in Figure 2, during the IA cytotoxicity assays, in the different cell lines, more morphological changes and a reduction in cell viability in the cancer cell lines were evident in comparation to the nontumorigenic cells. At concentrations of 5–7 μg/mL of IA, between 30–50% of the breast cancer cells, a reduced size, a rounded shape, the loss of cell adherence, and cell death were evident, while, in the nontumorigenic MCF10A line cells, they began to look affected between 10–13 μg /mL. When the cancer cells were treated with 12 μg/mL, practically all of them were found dead; however, in the MCF10A cells, even at 18 µg/mL, 15% of the cells were without alteration, and they seemed viable. As an example, changes in the MDA-MB-231 cancer cells are shown and are compared to MCF10A. Therefore, the specificity of IA towards the breast cancer cells was high, and at a concentration of 7 μg/mL, the effect upon nontumorigenic cells was minimal. Furthermore, its use as an antitumor treatment regardless of the molecular type of cancer promises to be safer (Figure 2).

### 2.3. Selectivity Index (SI) of IA

To confirm the data observed in the cell culture, the selectivity index (SI) was calculated. The SI indicates the selectivity of a certain compound against cancer cells but not against nontumorigenic cells. The greater the magnitude of the SI, the higher its preference against tumor cells is [13]. The estimate showed that IA had the highest selectivity towards the cancer cells (range of 1.73–2.32) compared to DEDi (1.3–1.6) and the drug doxorubicin (0.5–1.26) (Table 2). This supports that IA is a compound with important selectivity towards breast cancer cells that is not affected by the molecular type.

### 2.4. Effect of IA upon Glycolytic Enzymes

**Figure 3:**
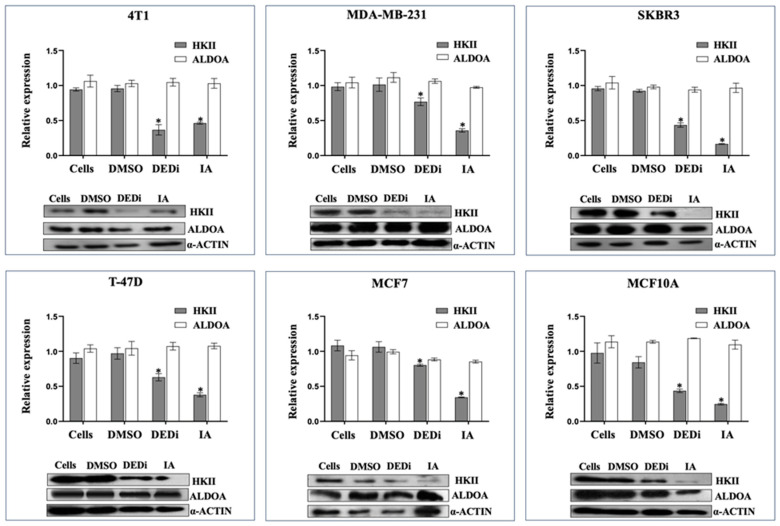
Expression of key glycolytic enzymes HKII (hexokinase II) and ALDOA (fructose-bisphosphate aldolase A) examined through Western blotting. The graphs show breast cancer cell lines 4T1, MDA-MB-231, SK-BR-3, T-47D, and MCF7 and the nontumorigenic cell line MCF10A. Cells were treated with IC_50_ of DEDi and IA (for 24 h and triplicate assays). Abbreviations: DMSO (dimethyl sulfoxide). * = Significant difference.

To identify the effect of IA upon glycolytic enzymes, modifications in the expression of hexokinase II (HKII), aldolase A (ALDOA), and lactate dehydrogenase (LDH, data not show) were analyzed through Western blotting. Each cell line was treated over 24 h with the CC_50_ for IA previously determined. For the cancer cell line 4T1, it was 6.2 µg/mL; for MDA-MB-231, it was 7.6 µg/mL; for SK-BR-3, it was 5.6 µg/mL; for T-47D, it was 7.8 µg/mL; for MCF-7, it was 6.8 µg/mL; and for the nontumorigenic MCF10A, it was 13 µg/mL. The results showed that, in the presence of IA, the expression of HKII decreased significantly after comparing the proportions obtained against the expression of actin in all the cell lines compared to the cells treated with DMSO and the cells without treatment (Figure 3).

### 2.5. Molecular Docking Studies of IA with HKII

**Figure 4:**
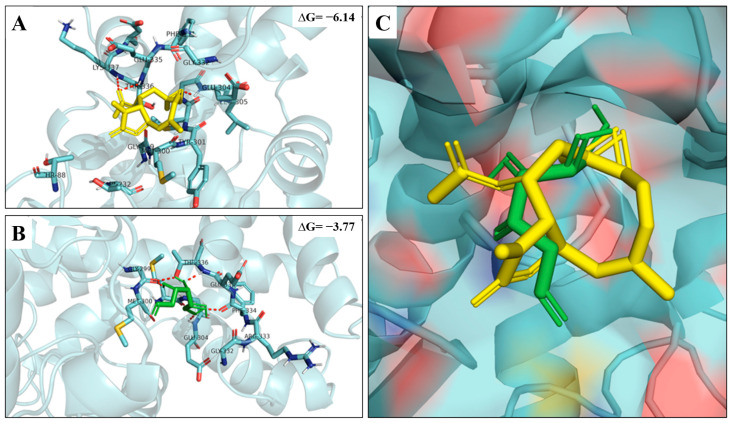
Results of molecular docking on hexokinase II enzyme. (**A**) Interaction of IA against HKII and its binding site position (yellow); (**B**) interaction of 2-DG against HKII and its binding site position (green); and (**C**) superimposed poses of IA and 2-DG in the binding site bound to HKII.

To evaluate the existence of putative interaction between IA and HKII, molecular docking was carried out. The docking assays showed that IA bound directly to the HKII with a binding energy of −6.14 kcal/mol and with polar interactions in the Glu304, Thr336, and Lys337 residues (Table 3 and Figure 4A). To analyze if the binding of IA was sufficient to inhibit the expression of HKII, we compared an already known inhibitor of HKII with 2-Deoxyglucose (2-DG). The 2-DG interaction with HKII presented a binding energy of −3.77 kcal/mol and polar interactions with the Gly299, Glu304, Phe334, and Thr336 residues (Table 3 and Figure 4B). This analysis showed that IA binding to HKII was similar to inhibitor 2-DG binding (Figure 4C), so IA could be able to bind directly and inhibit HKII like 2-DG does.

### 2.6. Toxicoinformatic and Pharmaceutical Analysis of IA

A common problem in drug development is that new compounds show remarkable properties *in vitro*, but when their toxicological and pharmacokinetic properties are reviewed, they are not adequate for clinical use; therefore, it is necessary to perform approaches to the physiochemical and toxicological properties of the compounds and to consider if they have pharmacological potential [13,14]. In this context, we analyzed *in silico* the physicochemical and toxicological properties of IA to determine if it has pharmacological potential. Using toxicoinformatics tools, we predicted some of these properties for IA (Table 4).

## 3. Discussion

Despite the advances in targeted therapies for breast cancer, there is still no treatment that is effective for all subtypes or stages of breast cancer; accordingly, it is necessary to search for new agents with better anticancer activity. In the present study, we demonstrated that the compound incomptine A has cytotoxic activity against breast cancer cells *in vitro* as well as reduces the expression of the hexokinase II protein. As far as we know, this is the first approximation that suggests that the possible mechanism of action of IA upon tumor cells is through its binding to HKII. 

According to the criteria of the National Cancer Institute, a pure compound is considered active when its CC_50_ is less than 10 μg/mL [13]. In this case, IA meets the criteria of an active compound for tumor cell treatment since, on average, a concentration of 7 mg/mL is required for a significant CC_50_. In contrast, for nontumorigenic cells (MCF10A) IA has a moderate cytotoxic activity since its CC_50_ is 13 mg/mL, and, according to the criteria, if the compound has a CC_50_ greater than 10 μg/mL, it is considered moderately active [15]. Therefore, IA could indeed be used as a therapeutic agent, as, at its active concentration (on average, 7 mg/mL), it would not have a significant effect on nontumorigenic cells but would significantly affect breast cancer cells. On the other hand, for doxorubicin to provoke an IA-like effect, a much higher dose is required. Hence, in this way, IA can be considered more efficient. In addition, IA required similar amounts to have a cytotoxic effect on all the tested breast cancer cells regardless of the type of tumor (triple-negative, HER2, or luminal A). This suggests that it could be a compound that acts against any type of breast cancer cell.

Most of the drugs used against breast cancer have a toxic effect on both tumor cells and healthy cells, so, to avoid this toxicity on normal cells, the doses are reduced, which affects the response to treatment of tumor cells. Therefore, it is essential to find compounds with greater selectivity for tumor cells. In this context, IA can be considered to be a bioactive compound and to be nontoxic against normal cells because its selectivity index turned out to be in the range of 1.73 to 2.32 and because it has been reported that an SI greater than 1.0 indicates that a drug is more selective for tumor cells [16]. Meanwhile, doxorubicin was more toxic, and its SI was from 0.5 to 1.23.

Currently, the explored treatment options for breast cancer are focused on drugs that control cell proliferation and induce cell death; however, the side effects are usually severe [17]. Cancer cells enhance their glycolysis, producing lactate even in the presence of oxygen, and the glycolytic enzymes are generally overexpressed, which is an important step for promoting cancer cell survival, proliferation, chemoresistance, and dissemination [18]. Consequently, developing new therapies directed at a different target, such as glycolytic metabolism, would be advantageous to counteract cancer, the reprogramming of its metabolism to a mainly glycolytic one being one of the hallmarks of cancer cells. 

HKII, in addition to participating in glycolytic metabolism, is considered an apoptotic regulator since it has physical interactions with the mitochondrial membrane through its binding to VDAC1. HKII associates, through its N-terminal domains, with VDCA1, generating an antiapoptotic event by preventing the release of cytochrome c (Cyt c) [19]. In this context, when we analyzed the effect of incomptine A on the expression of the key enzymes of the glycolytic pathway, such as hexokinase II, aldolase, and lactate dehydrogenase, we found that only HKII showed a significant decrease in its expression and that the others remained stable; this leads us to suggest that the reduction in HKII did not affect the glycolytic pathway and therefore is another affected route in which HKII participates, which could indicate that the cytotoxic effect and mechanism of action of IA are directly related to the other function of HKII. 

Molecular docking demonstrated that IA has strong binding with HKII (−6.14 kcal/mol), and, in the *in silico* comparison with 2-DG (an already described inhibitor for HKII), we observed that both share practically the same binding site, mostly with the amino acid residues GLU304 and THR336, and even that 2-DG has a lower binding strength (−3.77 kcal/mol) than IA, which suggests that the binding of IA to HKII is direct and that this could be the reason for the decrease in the expression of HKII. Additionally, several naturally occurring compounds tested in anticancer studies downregulate HKII with interesting results, where apoptosis is induced. For example, quercetin was shown to inhibit the proliferation of liver cancer cells by decreasing the HKII protein level [20], and Chrysin triggers cell apoptosis in hepatocellular carcinoma cells by targeting HKII [21]. These observations lead us to assume that, like 2-DG, IA binds directly to HKII, causing its inhibition, and, therefore, its binding to VDAC1 does not take place, thus enabling the release of cytochrome c and tumor cell apoptosis. This hypothesis may be supported since it has been reported that, in *in vivo* models of breast cancer, 2-DG induced oxidative stress and apoptosis [22]. 

Through computer tools, we performed the prediction of the pharmaceutical and toxicological properties of IA. This analysis showed that IA is a molecule with an adequate pharmacokinetic profile since no mutagenic or tumorigenic effects were observed, and it complies with Lipinski’s Rule of Five, which determines if a chemical compound has properties that would make it a medicine that can be used in humans due its pharmacological or biological activity or by assessing drug similarity [23]. On the other hand, in a previous study, the acute oral toxicity of IA was evaluated in a murine model of lymphoma and was comparable with the drug methotrexate, so these predictions can be extrapolated to animals; however, all this needs to be confirmed in animal models of breast cancer and perhaps in future clinical studies [6].

**Figure 5:**
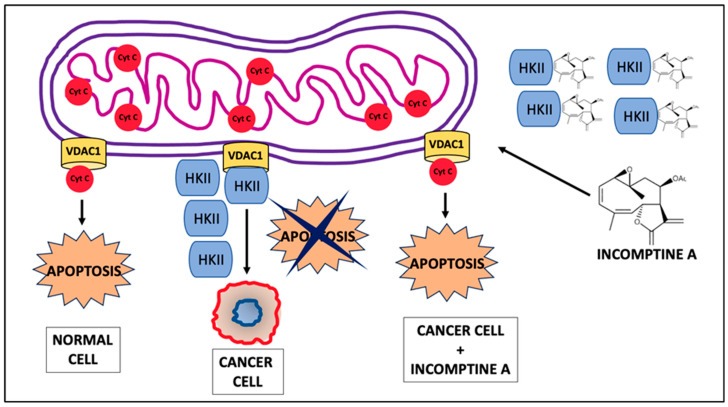
Proposed mechanism of action for IA in breast cancer cells. In breast cancer, KHII is overexpressed and bound to VDCA1, which prevents the presence of apoptosis. When cells are treated with IA, it binds to HKII, preventing the binding of HKII with VDAC1 and leaving the pore free with which apoptosis can be initiated.

Finally, given that, in cancer, the overexpression of HKII and its association with VDAC1 in the mitochondrial membrane of cancer cells inhibits apoptosis, we propose that, in breast cancer cells, the cytotoxic activity of IA is the consequence of its binding to HKII. IA-HKII binding provokes the release of VDAC1, which, in turn, will allow the exit of Cyt c, and apoptosis will occur (Figure 5). This first approximation will give us the guidelines to carry out more specific analyses and corroborate this hypothesis in *in vivo* models.

## 4. Materials and Methods

### 4.1. Collection and Identification of Decachaeta Incompta

*Decachaeta incompta* (D.C.) King and Robinson (Asteraceae) aerial parts were collected in Portillo Nejapa de Madero (16°36′00″ N, 95°59′00″ O), state of Oaxaca, Mexico. The plant was botanically authenticated by M Sc Abigail Aguilar, and a specimen (voucher 15311) was deposited at the Medicinal Herbarium IMSSM of the Instituto Mexicano del Seguro Social (IMSS).

### 4.2. Incomptine A

DEDi and IA were donated by Dr. Fernando Calzada, which were isolated according to the method described by Calzada et al., 2009 [24]. The identification of incomptine A was made through nuclear magnetic resonance with an authentic sample with a purity of 99%.

### 4.3. Cell Lines

4T1 (mouse breast cancer cell line, cat CRL-2539), MDA-MB-231 (triple-negative human breast adenocarcinoma cell line, cat HTB-26), SK-BR-3 (HER2 breast cancer, cat HTB-30), T-47D (luminal A subtype breast cancer cell line, cat HTB-133), MCF7 (luminal A subtype breast cancer cell line, cat HTB-22), and MCF10A (human breast epithelial cell line, cat CRL-10317) were acquired from the bank of the American Type Culture Collection (ATCC) through exclusive distributors. 4T1 and T-47D cell lines were maintained in RPMI-1640 medium (GIBCO cat 11875093) with 100 µg/mL of penicillin/streptomycin (GIBCO 15140148) supplemented with 10% fetal bovine serum (FBS) (GIBCO 26140079). MDA-MB-231, SK-BR-3, and MCF7 cell lines were maintained in Dulbecco’s Modified Eagle Medium (DMEM) (GIBCO 12491023) with 4.5 g/L of glucose and L-glutamine supplemented with 10% whey fetal bovine serum (FBS) and 1% antibiotic. Finally, MCF10A was maintained in DMEM/F12 medium (Biowest L0093-500) supplemented with 5% fetal horse serum (FHS) (GIBCO 16050130), 10 µg/mL of insulin, 0.5 µg/mL of hydrocortisone, 20 ng/mL of endothelial growth factor (EGF), and 1% antibiotic. All cell lines were incubated in a humid atmosphere at 37 °C and 5% CO_2_.

### 4.4. Chemicals

Doxorubicin was purchased from Zurich pharma, and dimethyl sulfoxide (DMSO) (Sigma–Aldrich cat 276855), L-glutamine, insulin, hydrocortisone, and endothelial growth factor were purchased from Sigma–Aldrich, Chemie GmbH, Taufkirchen, Germany. 

### 4.5. In Vitro Cytotoxicity Assay

Cell cytotoxicity was measured using the WST-1 assay, which is based on the reduction of tetrazolium salt, WST-1, to formazan by cellular mitochondrial dehydrogenase [25]. Cell proliferation was assessed using the Quick Cell Proliferation Kit II (Abcam, Cambridge, UK, Cat. No. ab65475) according to the manufacturer’s protocol. It consisted of seeding 96-well plates with each of cell lines at a concentration of 5 × 10^4^ cells per well in a final volume of 100 µL/well of culture medium. After 24 h of incubation, they were treated with different concentrations of DMSO 1%, DEDi (from 25 μg/mL up to 125 μg/mL), and IA (from 5 μg/mL up to 18 μg/mL); DEDi and IA were dissolved in DMSO. Each concentration was evaluated in triplicate. Untreated cells were used as negative controls, and cells with doxorubicin concentrations (20–150 μg/mL) were used as positive controls. After 24 h, cell viability was evaluated by incubating cells for 2 h with WST-1 reagent and by adding 10 µL to each pellet. At the end of the time, the cells were shaken well for one minute, and cell viability was determined by measuring absorbance at 440 nm with a microplate reader. The selectivity index (SI) was calculated as the average of the IC_50_ value of the normal cell line (MCF-10A) divided by the IC50 value of each cancer cell line obtained in each independent experiment.

### 4.6. Cell Morphology Analysis

Morphological alterations and cell damage were qualitatively investigated using an inverted phase contrast fluorescence microscope (Olympus CKX41), and the photos were taken with a digital camera.

### 4.7. Western Blotting

After the treatment of DEDi and IA, breast cancer and 10A cells were lysed with RIPA lysis buffer containing protease cocktail (Santa Cruz Biotechnology, Dallas, TX, USA) on ice for 15 min. The cell lysate was harvested and centrifugated at 10,000 × *g* for 10 min, and the supernatant was collected. The protein concentrations were determined with Quick Start™ Bradford Protein Assay Kit 1 (5000201). Thirty micrograms per sample were subjected to SDS-PAGE and were then transferred onto polyvinylidene difluoride membranes (Merck IPVH00010). After blocking the nonspecific binding site on the membrane with 5% nonfat milk solution, they were incubated with the following protein-specific antibodies overnight at 4 °C: mouse anti-HKII (1:2500, Santa Cruz Biotechnology, sc-130358), mouse anti-ALDOA (1:2500, Santa Cruz Biotechnology, sc-390733), and mouse anti-LDH (1:2500, Santa Cruz Biotechnology, sc-133123). Donkey antimouse antibody conjugated to horseradish peroxidase (HRP) (1:2500, abcam, ab6820) was used for secondary detection, and samples were visualized with chemiluminescence reagent (Thermo Fisher Scientific, Waltham, MA, USA).

### 4.8. Molecular Docking Studies

The chemical structures of incomptine A (IA) (CID: 118707242) and 2-deoxyglucose (2-DG) (CID: 108223) were retrieved from the chemical library PubChem (https://pubchem.ncbi.nlm.nih.gov/) (accessed on 20 July 2023). These were optimized and submitted to energetic and geometrical minimization using the Avogadro software. The 3D structures involved in the glycolytic metabolism of cancer cells were retrieved from the Protein Data Bank (https://www.rcsb.org) (accessed on 20 July 2023) with the following accession codes: hexokinase II (PDB ID: 2NZT). The total molecules of water and ions that were not needed for catalytic activity were stripped to preserve the entire protein. All polar hydrogen atoms were added and ionized in a basic environment (pH = 7.4), and Gasteiger charges were assigned. The computed output topologies from the previous steps were used as input files for docking simulations. The molecular docking experiments were carried out using AutoDock 4.2 software. The grid was centered at the following coordinates: HXII (center x = −3.228, center y = 13.415, and center z = 16.595) with grid dimensions of 60 × 60 × 60 points. The Lamarckian genetic algorithm was employed as a scoring function with a randomized initial population of 100 individuals and a maximum number of energy evaluations of 1 × 107 cycles. The analysis of the interactions in the enzyme/inhibitor complex was visualized with PyMOL software (the PyMOL Molecular Graphics System, Ver 2.0, Schrödinger, LLC, NY, USA). The validation of molecular docking was carried out by redocking the cocrystallized ligand in the receptors. The lowest energy pose of the cocrystallized ligands was superimposed, and it was observed whether it maintained the same binding position. The RMSD were calculated, and a reliable range within 2 Å was reported.

### 4.9. Docking Validation Protocol

The validation of the molecular docking results was conducted by redocking the cocrystallized ligand in the receptor (PDB ID: 2NZT). The lowest energy pose of redocking and the cocrystallized ligands were superimposed, and it was observed whether it gained the same position; additionally, its RMSD was calculated between these two superimposed ligands. The RMSD was within a reliable range of 2 Å.

### 4.10. In Silico Toxicology and Pharmaceutical Properties

There are different free access programs that are used to determine if a molecule possesses some pharmacological potential and possible toxicological risks through human consumption. The two-dimensional and three-dimensional structures of incomptine A were obtained from Pubchem CID 118707242. The simplified line entry system of molecular entry (SMILES) of incomptine A was used in the free access software Molinspiration, SwissAdme, and ADMETsar to evaluate the physicochemical properties.

### 4.11. Statistical Analysis

The results are expressed as mean ± standard error of the mean (SEM). Statistical analysis of the data was performed using one-way ANOVA with a value of *p* < 0.05 to establish a significant difference between the study groups. The CC50 was calculated through linear interpolation of the percentage mortality values for each concentration. All analyses were performed using Graph Pad Prism version 8 (GraphPad Software Inc., La Jolla, CA, USA).

## 5. Conclusions

IA can be considered to be more efficient in its cytotoxic activity and selectivity for breast cancer cells, and it can act in a similar way regardless of the cancer subtype (triple-negative, HER2, or luminal A); therefore, it is a compound that could be used against almost any subtype of breast cancer. According to the prediction of Lipinski’s Rule of Five, the physicochemical and toxicological properties of IA show that it is a molecule with an adequate pharmacokinetic profile, that it would not present mutagenic or tumorigenic effects, and that it could potentially be used in *in vivo* studies and in the future in clinical studies. 

We suggest that the mechanism by which IA could be leading to the death of tumor cells is through its binding to HKII, which, in turn, no longer joins to VDAC1, thus allowing the resumption of apoptosis. However, more studies are needed to confirm this hypothesis.

## Figures and Tables

**Table 1:** Cytotoxic activity of IA, DEDi, and doxorubicin against breast cancer and non-tumorigenic cell lines.

	Cytotoxic Concentration 50 (CC_50_) * in µg/mL					
	4T1	MDA-MB-231	SK-BR-3	T-47D	MCF7	MCF10A
IA	6.209 ± 0.38	7.58 ± 0.25	5.64 ± 0.09	7.83 ± 0.056	6.844 ± 0.25	13.12 ± 0.35
DEDi	54.89 ± 0.50	51.51 ± 0.22	56.735 ± 1.24	44.83 ± 1.40	44.18 ± 2.31	71.79 ± 1.88
Doxorubicin	76.36 ± 3.030	32.53 ± 0.26	67.75 ± 2.61	39.58 ± 0.33	72.61 ± 2.25	41.11 ± 1.39

* CC_50_ is defined as the treatment concentration at which a 50% reduction in cell turnover was demonstrated. Data are represented as means ± S.E.M. (*n* = 3); they were analyzed using GraphPad Prism. Abbreviations: 4T1 (triple-negative mouse breast cancer), MDA-MB-231 (triple-negative breast cancer), SK-BR-3 (HER2 breast cancer), T-47D (luminal A breast cancer), MCF7 (luminal A breast cancer), MCF10A (nontumorigenic breast cell line), IA (incomptine A), and DEDi (dichloromethane extract of the aerial parts of *Decachaeta incompta*).

**Table 2:** Selectivity index of IA, DEDi, and doxorubicin against breast cancer cell lines.

	Cell Lines				
	4T1	MDA-MB-231	SK-BR-3	T-47D	MCF7
Incomptine A	2.11	1.73	2.32	1.77	1.91
DEDi	1.30	1.39	1.26	1.60	1.62
Doxorubicin	0.5	1.26	0.60	1.03	0.5

**Table 3:** Interactions of IA and 2-Deoxyglucose (2-DG) with HKII.

	Incomptine A (IA)			2-Deoxyglucose (2-DG)			
Glycolytic Enzyme	ΔG (kcal/mol)	H-Binding Residues	Nonpolar Interactions	ΔG (kcal/mol)	H-Binding Residues	Nonpolar Interactions	RMSD
HKII (2NZT)	−6.14	GLU304 THR336 LYS337	GLY299 TYR301 LEU305 GLY332 PHE334 GLU335	−3.77	GLY299 GLU304 PHE334 THR336	MET300 ARG333 GLU335	0.94

**Table 4:** Physicochemical and toxicological properties of IA (prediction properties ^a^).

Physicochemical	
Chemical formula	C17H20O5
Molecular weight	304.34 g/mol
Topological polar surface area (TPSA)	65.14 A
Lipophilicity (LogP)	1.62
Water solubility (LogS)	−2.69
Number of H donors	0
Number of H-bond acceptors	5
Rotatable bonds	2
Pharmacokinetic	
Gastrointestinal absorption	HIGH
Blood–brain barrier permeability	YES
P-glycoprotein substrate	NO
Cytochrome P450 1A2 inhibitor	NO
Cytochrome P450 2C19 inhibitor	NO
Cytochrome P450 2C9 inhibitor	NO
Cytochrome P450 2D6 inhibitor	NO
Cytochrome P450 3A4 inhibitor	NO
Druglikeness	
Lipinski	YES; 0 Violations
Ghose	YES
Veber	YES
Egan	YES
Muegge	YES
Toxicoinformatic	
Toxicity class *	CLASS III
Mutagenic	NONE
Tumorigenic	NONE

^a^ Predictions were based on Molinspiration, SwissADME, and ADMETsar web servers, and they were * based on toxic classes defined according to the globally harmonized system of classification of labeling of chemicals, where Category I is fatal if swallowed (LD_50_ < 5 mg/kg), Category II is fatal if swallowed (LD_50_ < 50 mg/kg), Category III is toxic if swallowed (LD_50_ < 300 mg/kg), Category IV is harmful if swallowed (LD_50_ < 2000 mg/kg), Category V may be harmful if swallowed (LD_50_ < 5000 mg/kg), and Category VI is nontoxic (LD_50_ > 5000 mg/kg).

## Data Availability

The data generated in this manuscript can be requested from the Ph.D. Rosa María Ordoñez-Razo by email (romaorr@yahoo.com.mx).
